# Supplementary material for: Anserine/Carnosine-Rich Extract from Thai Native Chicken Suppresses Melanogenesis via Activation of ERK Signaling Pathway
Source: Molecules. 2022 Nov 2;27(21):7440. doi: 10.3390/molecules27217440 (PMC9659164; doi:10.3390/molecules27217440)
Supplement: Supplementary file 1 [file molecules-27-07440-s001.zip › molecules-1906324-Supplementary.pdf]

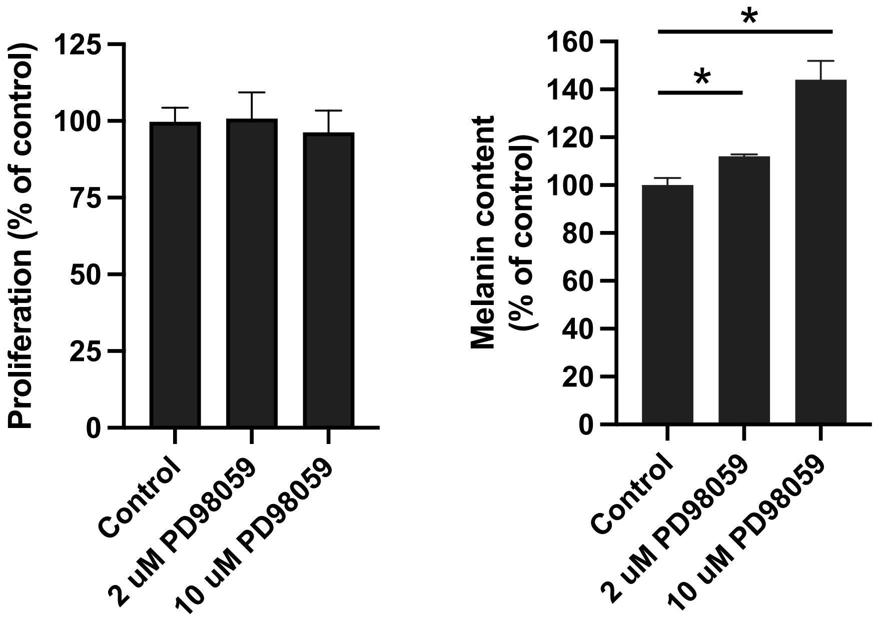


**Supplementary Figure S1.** Effect of PD98059 (MEK inhibitor) on MNT-1 proliferation (left) and melanin production (right) * = significant different ($p < 0.05$)
